# Supplementary material for: New Dothideomycetes from Freshwater Habitats in Spain
Source: J Fungi (Basel). 2021 Dec 20;7(12):1102. doi: 10.3390/jof7121102 (PMC8705806; doi:10.3390/jof7121102)
Supplement: Supplementary file 1 [file jof-07-01102-s001.zip › 2.jof-1508898-supplementary.pdf]

Table S1. Fungal taxa and their nucleotide sequences of the molecular markers used to build the phylogenetic trees.

Taxon	Strain	GenBank Accession No.			
		ITS	*tub2*	*rpb2*	*tef1*
Acericola italica	MFLUCC:13-0609	NR_156344			
Didymella brevipilosa	**FMR 17415**	**OU612373**	**OU612358**	**OU612359**	
Didymella ellipsoidea	CGMCC3.18350	KY742060	KY742302	KY742145	
Didymella exigua	CBS 183.55	GU237794	GU237525	EU874850	
Didymella macrostoma	CBS 223.69	GU237801	GU237623	KT389608	
Didymella pteridis	CBS 379.96	KT389504	KT389801	KT389624	
Didymella rumicicola	CBS 683.79	KT389503	KT389800	KT389622	
Didymella subrosea	CBS 733.79	NR_170787	MT005643	MT018174	
Didymella viburnicola	CBS 523.73	GU237879	GU237667	MH872477	
Epicoccum nigrum	CBS 173.73	FJ426996	FJ427107	KT389631	
Epicoccum ovisporum	CBS 180.80	FJ427068	FJ427174	LT623252	
Epicoccum plurivorum	CBS 558.81	GU237888	GU237647	KT389634	
Heterophoma adonidis	CBS 114309	MH862963	KT389803	KT389637	
Heterophoma nobilis	CBS 507.91	NR_170721	GU237603	KT389638	
Heterophoma polypusiformis	**FMR17837**	**OU612367**	**OU600611**	**OU600610**	
Heterophoma poolensis	CBS 113.20	MH854684	GU237638	MT018056	

Heterophoma sylvatica	CBS 874.97	GU237907	GU237662	MT018052	
Heterophoma verbascicola	CGMCC 3.18364	NR_158268	GU237650	KY742187	
Heterophoma verbasci-densiflori	CBS 449.81	MN973474	MT005573	MT018049	
Jeremyomyces labinae	CBS:144647	NR_163362	MK442733	MK442665	**OU600603**
Neoconiothyrium hakeae	CPC 27620	NR_154839	KY173600	KY173583	
Neoconiothyrium multiporum	CBS 353.65	MH858605			
Neoconiothyrium persooniae	CBS 143175	MG386041			
Neoconiothyrium sp.	**FMR 17669**	**OU641117**	**OU641013**	**OU641014**	
Neoconiothyrium viticola	CPC 36397	MN562123		MN556804	
Neophaeosphaeria agaves	CBS:136429	NR_137833			
Neophaeosphaeria filamentosa	CBS 102202	JF740259		GU357803	GU349084
Paraboeremia adianticola	CBS 187.83	GU237796	GU237576	KP330401	
Paraboeremia camelliae	CGMCC3.18106	KX829034	KX829058	KX829050	
Paraboeremia clausa	**FMR 18597**	**OU612369**	**OU600598**	**OU600597**	
Paraboeremia clausa	**FMR 18598**	**OU612371**	**OU600600**	**OU600599**	
Paraboeremia litseae	CGMCC3.18109	KX829029	KX829053	KX829045	
Paraboeremia oligotrophica	CGMCC3.18111	KX829031	KX829055	KX829047	
Paraboeremia putaminum	CBS 299.39	MN823454	MN824628	MN824480	
Paraboeremia rekkeri	CBS 144955	MN823511	MN824685	MN824537	

Paraboeremia selaginellae	CBS 122.93	GU237762	GU237656	LT623255	
Paraboeremia taiwanensis	NTUCC 17-013	MK840826	MK839232	MK839234	
Paraboeremia truiniorum	CBS 144952	MN823495	MN824669	MN824521	
Paraphoma aquatica	**FMR 16956**	**OU612361**	**OU612355**	**OU612357**	**OU612356**
Paraphoma dioscoreae	CBS 135100	KF251167	KF252662		
Paraphoma chrysanthemicola	CBS 172.70	KF251165	KF252660		
Paraphoma fimeti	UTHSC:DI16-296	LT796872	KF252665	LT797032	LT797112
Paraphoma melnikiae	MF-9.88	MG764063	MG779456	MG779466	
Paraphoma radicina	UTHSC:DI16-209	LT796835	KF252667	LT796995	LT797075
Paraphoma rhaphiolepidis	CBS 142524	KY979758	KY979924	KY979851	
Paraphoma vinacea	UMPV004	KU176887	KU176895		
Phaeosphaeria breonadiae	CBS 141334	NR_155675			
Phaeosphaeria caricicola	CBS 603.86	KF251182	KF252676		
Phaeosphaeria fructigena	**FMR 17808**	**OU612363**	**OU600608**	**OU600607**	**OU600609**
Phaeosphaeria glyceriae-plicatae	CBS 101261	MH862724			
Phaeosphaeria juncophila	CBS 575.86	AF439488			GU456283
Phaeosphaeria lutea	CBS 455.84	MH861760			
Phaeosphaeria norfolcia	CBS 593.86	MH861997			
Phaeosphaeria olivacea	MUTITA 2854	MG813228			

Phaeosphaeria parvula	CBS 605.86	MH862001			
Phaeosphaeriopsis glaucopunctata	MFLUCC:13-0220	KJ522473	KF252693		MG520918
Phaeosphaeriopsis triseptata	MFLUCC:13-0271	KJ522475		KJ522485	MG520919
Setomelanomma holmii	CBS 110217	KT389542		GU371800	GU349028
Vacuiphoma bulgarica	CBS 357.84				
Vacuiphoma oculihominis	UTHSC:DI16-308				
Xenophoma microspora	**FMR 17947**	**OU612365**	**OU600605**	**OU600604**	**OU600606**
Xenophoma puncteliae	CBS 128022	JQ238617	KP170711	**OU600601**	**OU600602**
Xenoseptoria neosaccardoi	CBS 120.43	KF251280	KF252761		

[1]**CBS**: Culture collection of the Westerdijk Biodiversity Institute, Utrech, The Netherlands; **CGMCC**: China General Microbiological Culture Collection Center; **FMR**: Facultat de Medicina, Reus, Spain; **CPC** :Culture Collection of Pedro Crous; **MFLUCC** : Mae Fah Luang University Culture Collection, Chiang Rai, Thailand ; **NTUCC**: National Taiwan University Culture Collection; **UMPV**: University of Melbourne, *Paraphoma vinacea* strain **UTHSC**, Fungus Testing Laboratory at the University of Texas Health Science Center, San Antonio, Texas, USA

[2]Strains studied by us are indicated in **bold**.

[T]Ex-type strain.

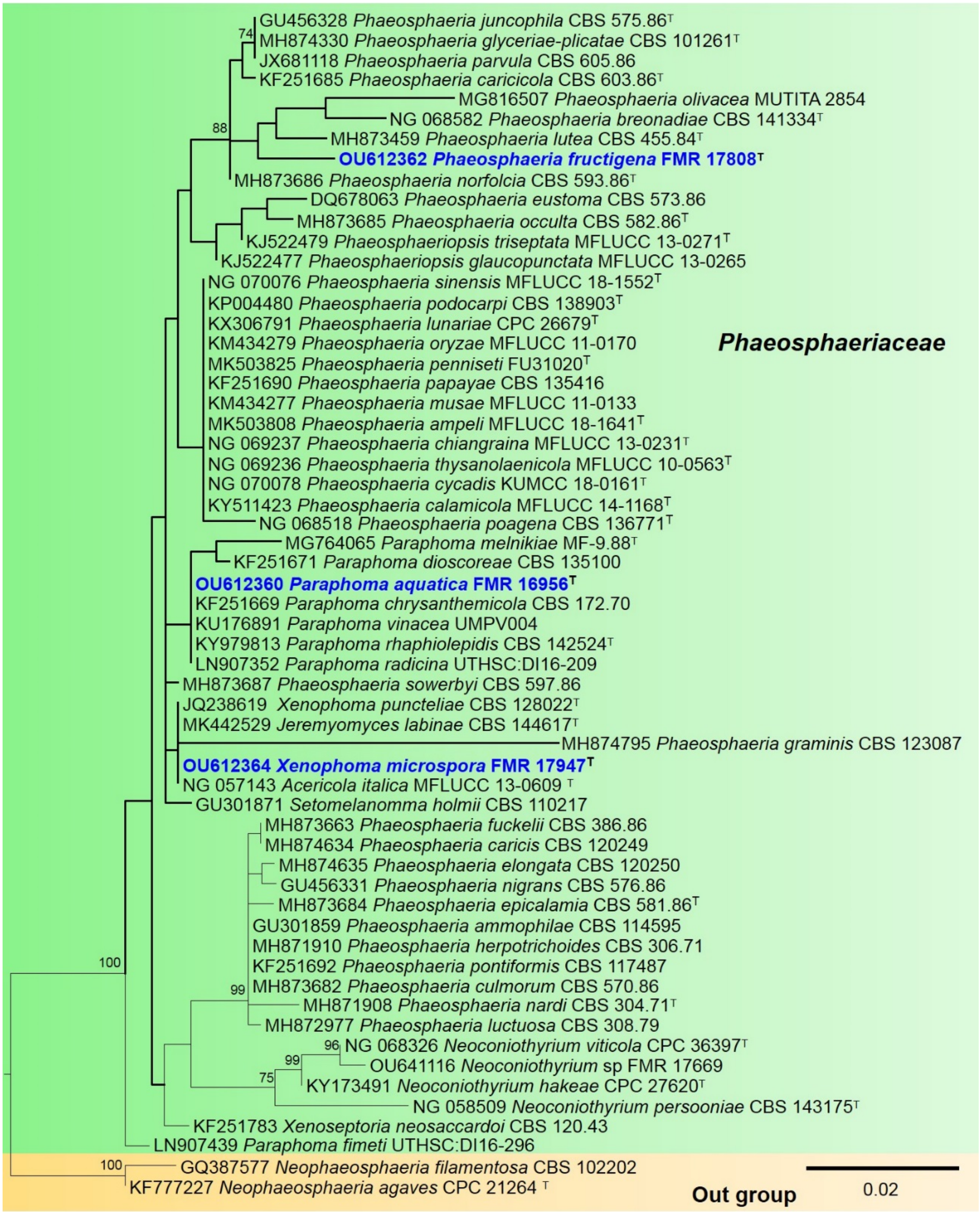

Figure S1 ML phylogenetics tree of *Phaeosphaeriacea* inferred from the LSU sequences of 59 strains. Support in nodes is indicated above by bootstrap values of 70% or highter. The tree was rooted with *Neophaeosphaeria agaves* CBS 136429 and *Neophaeosphaeria filamentosa* CBS 102202. Alignment length 713 b.p. Newly proposed taxa are given in blue. Type strains are indicated by a superscript T.
